# Supplementary material for: The Associations of cerebral blood flow and white matter hyperintensities with tau and amyloid-beta across the Alzheimer’s disease spectrum
Source: Brain Imaging Behav. 2026 Aug 5;20(4):117. doi: 10.1007/s11682-026-01187-x (PMC13442569; doi:10.1007/s11682-026-01187-x)
Supplement: Supplementary file 1 [file 11682_2026_1187_MOESM1_ESM.docx]

|  | **Adjusting for age, sex, APOE** ε4 and hypertension | | | | **Further adjusting for Aβ** | | | |
| --- | --- | --- | --- | --- | --- | --- | --- | --- |
| Regions | **β** | **CI lower** | **CI upper** | **P value** | **β** | **CI lower** | **CI upper** | **P value** |
| Banks STS | -0.250 | -0.454 | -0.046 | 0.017 | -0.243 | -0.463 | -0.023 | 0.031 |
| Caudal anterior cingulate | 0.011 | -0.245 | 0.266 | 0.935 | 0.065 | -0.208 | 0.338 | 0.637 |
| Caudal middle frontal | -0.252 | -0.437 | -0.068 | 0.008* | -0.234 | -0.433 | -0.035 | 0.021 |
| Cuneus | -0.139 | -0.308 | 0.031 | 0.108 | -0.112 | -0.302 | 0.079 | 0.248 |
| Entorhinal | -0.145 | -0.322 | 0.033 | 0.109 | -0.200 | -0.408 | 0.008 | 0.059 |
| Frontal pole | -0.021 | -0.240 | 0.198 | 0.850 | -0.020 | -0.253 | 0.212 | 0.863 |
| Fusiform | -0.273 | -0.475 | -0.072 | 0.008* | -0.313 | -0.543 | -0.082 | 0.008 |
| Inferior parietal | -0.348 | -0.536 | -0.159 | < 0.001* | -0.336 | -0.541 | -0.131 | 0.002 |
| Inferior temporal | -0.284 | -0.501 | -0.067 | 0.011* | -0.267 | -0.511 | -0.023 | 0.032 |
| Isthmus cingulate | -0.300 | -0.489 | -0.111 | 0.002* | -0.283 | -0.488 | -0.079 | 0.007 |
| Lateral occipital | -0.234 | -0.416 | -0.053 | 0.012* | -0.286 | -0.509 | -0.064 | 0.012 |
| Lateral orbitofrontal | -0.071 | -0.291 | 0.149 | 0.524 | -0.065 | -0.300 | 0.171 | 0.588 |
| Lingual | -0.076 | -0.249 | 0.097 | 0.383 | -0.065 | -0.273 | 0.143 | 0.537 |
| Medial orbitofrontal | -0.031 | -0.274 | 0.211 | 0.798 | -0.014 | -0.273 | 0.246 | 0.918 |
| Middle temporal | -0.327 | -0.543 | -0.111 | 0.003* | -0.315 | -0.554 | -0.075 | 0.011 |
| Paracentral | -0.026 | -0.251 | 0.198 | 0.817 | 0.012 | -0.231 | 0.255 | 0.921 |
| Parahippocampal | -0.164 | -0.368 | 0.039 | 0.112 | -0.208 | -0.447 | 0.031 | 0.087 |
| Pars opercularis | -0.175 | -0.369 | 0.019 | 0.076 | -0.175 | -0.381 | 0.031 | 0.096 |
| Pars orbitalis | -0.039 | -0.244 | 0.166 | 0.706 | -0.026 | -0.243 | 0.192 | 0.815 |
| Pars triangularis | -0.098 | -0.297 | 0.100 | 0.328 | -0.095 | -0.307 | 0.117 | 0.375 |
| Pericalcarine | -0.062 | -0.228 | 0.103 | 0.456 | -0.020 | -0.230 | 0.189 | 0.850 |
| Postcentral | -0.224 | -0.451 | 0.002 | 0.053 | -0.196 | -0.444 | 0.051 | 0.119 |
| Posterior cingulate | -0.195 | -0.404 | 0.014 | 0.067 | -0.162 | -0.391 | 0.067 | 0.164 |
| Precentral | -0.159 | -0.355 | 0.037 | 0.111 | -0.137 | -0.352 | 0.078 | 0.208 |
| Precuneus | -0.268 | -0.456 | -0.080 | 0.006* | -0.263 | -0.466 | -0.061 | 0.011 |
| Rostral anterior cingulate | 0.086 | -0.173 | 0.345 | 0.512 | 0.104 | -0.163 | 0.371 | 0.441 |
| Rostral middle frontal | -0.159 | -0.359 | 0.041 | 0.119 | -0.156 | -0.371 | 0.058 | 0.151 |
| Superior frontal | -0.122 | -0.334 | 0.091 | 0.259 | -0.112 | -0.343 | 0.118 | 0.336 |
| Superior parietal | -0.277 | -0.457 | -0.097 | 0.003* | -0.251 | -0.454 | -0.049 | 0.015 |
| Superior temporal | -0.203 | -0.417 | 0.010 | 0.061 | -0.183 | -0.416 | 0.050 | 0.122 |
| Supramarginal | -0.319 | -0.524 | -0.114 | 0.003* | -0.313 | -0.535 | -0.091 | 0.006 |
| Temporal pole | -0.222 | -0.440 | -0.005 | 0.045 | -0.270 | -0.515 | -0.025 | 0.031 |
| Transverse temporal | 0.038 | -0.165 | 0.241 | 0.711 | 0.029 | -0.181 | 0.239 | 0.782 |
| Frontal | -0.250 | -0.454 | -0.046 | 0.017 | -0.243 | -0.463 | -0.023 | 0.031 |
| Temporal | 0.011 | -0.245 | 0.266 | 0.935 | 0.065 | -0.208 | 0.338 | 0.637 |
| Occipital | -0.252 | -0.437 | -0.068 | 0.008* | -0.234 | -0.433 | -0.035 | 0.021 |
| Parietal | -0.139 | -0.308 | 0.031 | 0.108 | -0.112 | -0.302 | 0.079 | 0.248 |

**Supplementary Table 1 – Associations between cerebral blood flow and tau levels further adjusting APOE and hypertension.**
Banks STS- Banks of the superior temporal sulcus; CI – Confidence Interval; CI lower – Lower bound of the 95% Confidence Interval; CI upper – Upper bound of the 95% Confidence Interval. * indicates associations that survived false discovery rate correction.

|  | **Adjusting for age, sex, APOE** ε4 and hypertension | | | | **Further adjusting for Aβ change** | | | |
| --- | --- | --- | --- | --- | --- | --- | --- | --- |
| Regions | **β** | **CI lower** | **CI upper** | **P value** | **β** | **CI lower** | **CI upper** | **P value** |
| Banks STS | -0.174 | -0.322 | -0.027 | 0.021 | -0.191 | -0.365 | -0.016 | 0.033 |
| Caudal anterior cingulate | -0.033 | -0.144 | 0.079 | 0.564 | -0.037 | -0.173 | 0.099 | 0.595 |
| Caudal middle frontal | -0.154 | -0.291 | -0.016 | 0.029 | -0.221 | -0.390 | -0.052 | 0.011* |
| Cuneus | -0.169 | -0.338 | 0.000 | 0.050 | -0.115 | -0.314 | 0.085 | 0.256 |
| Entorhinal | -0.174 | -0.335 | -0.012 | 0.035 | -0.204 | -0.372 | -0.036 | 0.018* |
| Frontal pole | -0.009 | -0.120 | 0.102 | 0.871 | 0.010 | -0.118 | 0.139 | 0.874 |
| Fusiform | -0.261 | -0.437 | -0.086 | 0.004* | -0.277 | -0.471 | -0.082 | 0.006* |
| Inferior parietal | -0.340 | -0.503 | -0.177 | < 0.001* | -0.337 | -0.511 | -0.163 | < 0.001* |
| Inferior temporal | -0.267 | -0.446 | -0.087 | 0.004* | -0.277 | -0.474 | -0.079 | 0.006* |
| Isthmus cingulate | -0.294 | -0.459 | -0.129 | < 0.001* | -0.262 | -0.439 | -0.085 | 0.004* |
| Lateral occipital | -0.224 | -0.386 | -0.062 | 0.007* | -0.269 | -0.457 | -0.080 | 0.006* |
| Lateral orbitofrontal | -0.066 | -0.174 | 0.043 | 0.232 | -0.085 | -0.225 | 0.055 | 0.231 |
| Lingual | -0.151 | -0.325 | 0.024 | 0.090 | -0.183 | -0.381 | 0.015 | 0.070 |
| Medial orbitofrontal | -0.086 | -0.196 | 0.023 | 0.123 | -0.117 | -0.257 | 0.023 | 0.099 |
| Middle temporal | -0.316 | -0.494 | -0.138 | < 0.001* | -0.312 | -0.504 | -0.121 | 0.002* |
| Paracentral | -0.082 | -0.183 | 0.018 | 0.107 | -0.065 | -0.186 | 0.056 | 0.291 |
| Parahippocampal | -0.205 | -0.381 | -0.028 | 0.023 | -0.153 | -0.364 | 0.059 | 0.157 |
| Pars opercularis | -0.155 | -0.278 | -0.032 | 0.014* | -0.141 | -0.290 | 0.009 | 0.065 |
| Pars orbitalis | -0.075 | -0.219 | 0.069 | 0.304 | -0.067 | -0.232 | 0.097 | 0.419 |
| Pars triangularis | -0.100 | -0.235 | 0.035 | 0.145 | -0.067 | -0.212 | 0.078 | 0.361 |
| Pericalcarine | -0.136 | -0.303 | 0.030 | 0.108 | -0.154 | -0.372 | 0.063 | 0.162 |
| Postcentral | -0.211 | -0.410 | -0.011 | 0.039 | -0.042 | -0.153 | 0.068 | 0.451 |
| Posterior cingulate | -0.196 | -0.376 | -0.015 | 0.034 | -0.069 | -0.210 | 0.072 | 0.332 |
| Precentral | -0.102 | -0.205 | 0.001 | 0.051 | -0.109 | -0.248 | 0.031 | 0.126 |
| Precuneus | -0.277 | -0.437 | -0.118 | < 0.001* | -0.241 | -0.413 | -0.068 | 0.007* |
| Rostral anterior cingulate | -0.067 | -0.166 | 0.031 | 0.180 | -0.059 | -0.183 | 0.065 | 0.347 |
| Rostral middle frontal | -0.131 | -0.257 | -0.005 | 0.042 | -0.116 | -0.265 | 0.033 | 0.126 |
| Superior frontal | -0.119 | -0.229 | -0.008 | 0.036 | -0.090 | -0.222 | 0.043 | 0.182 |
| Superior parietal | -0.272 | -0.434 | -0.110 | 0.001* | -0.261 | -0.437 | -0.085 | 0.004* |
| Superior temporal | -0.211 | -0.390 | -0.031 | 0.022 | -0.156 | -0.341 | 0.028 | 0.095 |
| Supramarginal | -0.319 | -0.489 | -0.149 | < 0.001* | -0.298 | -0.485 | -0.112 | 0.002* |
| Temporal pole | -0.201 | -0.393 | -0.009 | 0.041 | -0.251 | -0.452 | -0.049 | 0.015* |
| Transverse temporal | -0.021 | -0.119 | 0.077 | 0.668 | -0.044 | -0.173 | 0.086 | 0.505 |
| Frontal | -0.174 | -0.322 | -0.027 | 0.021 | -0.191 | -0.365 | -0.016 | 0.033 |
| Temporal | -0.033 | -0.144 | 0.079 | 0.564 | -0.037 | -0.173 | 0.099 | 0.595 |
| Occipital | -0.154 | -0.291 | -0.016 | 0.029 | -0.221 | -0.390 | -0.052 | 0.011* |
| Parietal | -0.169 | -0.338 | 0.000 | 0.050 | -0.115 | -0.314 | 0.085 | 0.256 |

**Supplementary Table 2 – Longitudinal Associations between Changes in Regional Cerebral Blood Flow and Changes in Tau Levels, Further Adjusted for Hypertension Category and APOE ε4 Status**

Banks STS - Banks of the superior temporal sulcus; CI – Confidence Interval; CI lower – Lower bound of the 95% Confidence Interval; CI upper – Upper bound of the 95% Confidence Interval. * indicates associations that survived false discovery rate correction.

|  | **Adjusting for age, sex, APOE** ε4 and hypertension | | | | **Further adjusting for Aβ** | | | |
| --- | --- | --- | --- | --- | --- | --- | --- | --- |
| Regions | **β** | **CI lower** | **CI upper** | **P value** | **β** | **CI lower** | **CI upper** | **P value** |
| Banks STS | -0.254 | -0.454 | -0.054 | 0.013 | -0.239 | -0.454 | -0.023 | 0.030 |
| Caudal anterior cingulate | 0.000 | -0.252 | 0.253 | 0.998 | 0.055 | -0.215 | 0.324 | 0.688 |
| Caudal middle frontal | -0.246 | -0.428 | -0.063 | 0.009* | -0.215 | -0.411 | -0.019 | 0.032 |
| Cuneus | -0.122 | -0.284 | 0.040 | 0.137 | -0.107 | -0.290 | 0.075 | 0.245 |
| Entorhinal | -0.156 | -0.366 | 0.054 | 0.144 | -0.121 | -0.367 | 0.125 | 0.332 |
| Frontal pole | -0.015 | -0.244 | 0.214 | 0.897 | -0.006 | -0.248 | 0.237 | 0.962 |
| Fusiform | -0.227 | -0.453 | -0.001 | 0.049 | -0.223 | -0.482 | 0.036 | 0.091 |
| Inferior parietal | -0.303 | -0.486 | -0.120 | 0.001* | -0.300 | -0.499 | -0.102 | 0.003 |
| Inferior temporal | -0.250 | -0.475 | -0.025 | 0.030 | -0.213 | -0.466 | 0.039 | 0.097 |
| Isthmus cingulate | -0.258 | -0.451 | -0.065 | 0.009* | -0.222 | -0.430 | -0.014 | 0.037 |
| Lateral occipital | -0.185 | -0.366 | -0.004 | 0.045 | -0.252 | -0.474 | -0.030 | 0.026 |
| Lateral orbitofrontal | -0.106 | -0.332 | 0.119 | 0.352 | -0.075 | -0.317 | 0.167 | 0.542 |
| Lingual | -0.057 | -0.233 | 0.118 | 0.520 | -0.042 | -0.254 | 0.169 | 0.692 |
| Medial orbitofrontal | -0.082 | -0.339 | 0.176 | 0.531 | -0.048 | -0.326 | 0.229 | 0.731 |
| Middle temporal | -0.294 | -0.509 | -0.079 | 0.008* | -0.268 | -0.506 | -0.030 | 0.028 |
| Paracentral | -0.034 | -0.256 | 0.187 | 0.758 | 0.013 | -0.226 | 0.252 | 0.915 |
| Parahippocampal | -0.143 | -0.359 | 0.073 | 0.193 | -0.086 | -0.342 | 0.170 | 0.509 |
| Pars opercularis | -0.173 | -0.372 | 0.027 | 0.090 | -0.149 | -0.361 | 0.062 | 0.165 |
| Pars orbitalis | -0.055 | -0.266 | 0.156 | 0.607 | -0.023 | -0.247 | 0.200 | 0.836 |
| Pars triangularis | -0.105 | -0.297 | 0.087 | 0.281 | -0.087 | -0.292 | 0.118 | 0.402 |
| Pericalcarine | -0.024 | -0.187 | 0.139 | 0.770 | 0.012 | -0.195 | 0.219 | 0.908 |
| Postcentral | -0.202 | -0.424 | 0.021 | 0.076 | -0.149 | -0.391 | 0.094 | 0.227 |
| Posterior cingulate | -0.206 | -0.419 | 0.007 | 0.057 | -0.152 | -0.385 | 0.081 | 0.199 |
| Precentral | -0.170 | -0.362 | 0.022 | 0.083 | -0.122 | -0.332 | 0.087 | 0.250 |
| Precuneus | -0.251 | -0.438 | -0.063 | 0.009* | -0.234 | -0.435 | -0.032 | 0.023 |
| Rostral anterior cingulate | 0.071 | -0.192 | 0.334 | 0.594 | 0.108 | -0.166 | 0.381 | 0.438 |
| Rostral middle frontal | -0.167 | -0.360 | 0.027 | 0.090 | -0.152 | -0.359 | 0.055 | 0.148 |
| Superior frontal | -0.127 | -0.339 | 0.085 | 0.237 | -0.106 | -0.337 | 0.124 | 0.363 |
| Superior parietal | -0.247 | -0.425 | -0.069 | 0.007* | -0.209 | -0.408 | -0.009 | 0.040 |
| Superior temporal | -0.196 | -0.407 | 0.016 | 0.069 | -0.165 | -0.395 | 0.066 | 0.159 |
| Supramarginal | -0.304 | -0.503 | -0.106 | 0.003* | -0.288 | -0.504 | -0.073 | 0.009 |
| Temporal pole | -0.102 | -0.343 | 0.140 | 0.406 | -0.059 | -0.331 | 0.213 | 0.668 |
| Transverse temporal | -0.060 | -0.260 | 0.140 | 0.554 | -0.050 | -0.257 | 0.158 | 0.637 |
| Frontal | -0.254 | -0.454 | -0.054 | 0.013 | -0.239 | -0.454 | -0.023 | 0.030 |
| Temporal | 0.000 | -0.252 | 0.253 | 0.998 | 0.055 | -0.215 | 0.324 | 0.688 |
| Occipital | -0.246 | -0.428 | -0.063 | 0.009* | -0.215 | -0.411 | -0.019 | 0.032 |
| Parietal | -0.122 | -0.284 | 0.040 | 0.137 | -0.107 | -0.290 | 0.075 | 0.245 |

**Supplementary Table 3 – Associations between partial volume corrected cerebral blood flow and tau levels.**
Banks STS- Banks of the superior temporal sulcus; CI – Confidence Interval; CI lower – Lower bound of the 95% Confidence Interval; CI upper – Upper bound of the 95% Confidence Interval. * indicates associations that survived false discovery rate correction.

|  | **Adjusting for age, sex, APOE** ε4 and hypertension | | | | **Further adjusting for Aβ** | | | |
| --- | --- | --- | --- | --- | --- | --- | --- | --- |
| Regions | **β** | **CI lower** | **CI upper** | **P value** | **β** | **CI lower** | **CI upper** | **P value** |
| Banks STS | -0.285 | -0.439 | -0.131 | < 0.001* | -0.229 | -0.397 | -0.061 | 0.008* |
| Caudal anterior cingulate | -0.088 | -0.243 | 0.068 | 0.267 | -0.066 | -0.234 | 0.102 | 0.439 |
| Caudal middle frontal | -0.229 | -0.374 | -0.084 | 0.002* | -0.208 | -0.371 | -0.046 | 0.012 |
| Cuneus | -0.165 | -0.301 | -0.029 | 0.018* | -0.128 | -0.278 | 0.023 | 0.097 |
| Entorhinal | -0.093 | -0.258 | 0.071 | 0.264 | -0.142 | -0.333 | 0.050 | 0.145 |
| Frontal pole | -0.036 | -0.204 | 0.131 | 0.671 | -0.024 | -0.206 | 0.158 | 0.793 |
| Fusiform | -0.163 | -0.324 | -0.002 | 0.047 | -0.196 | -0.383 | -0.010 | 0.039 |
| Inferior parietal | -0.323 | -0.469 | -0.177 | < 0.001* | -0.298 | -0.461 | -0.134 | < 0.001* |
| Inferior temporal | -0.172 | -0.331 | -0.013 | 0.034 | -0.193 | -0.378 | -0.009 | 0.040 |
| Isthmus cingulate | -0.213 | -0.362 | -0.064 | 0.005* | -0.203 | -0.366 | -0.039 | 0.015 |
| Lateral occipital | -0.192 | -0.342 | -0.043 | 0.012* | -0.225 | -0.403 | -0.047 | 0.014 |
| Lateral orbitofrontal | -0.106 | -0.264 | 0.053 | 0.189 | -0.100 | -0.275 | 0.076 | 0.264 |
| Lingual | -0.107 | -0.253 | 0.038 | 0.148 | -0.067 | -0.236 | 0.101 | 0.432 |
| Medial orbitofrontal | -0.083 | -0.246 | 0.080 | 0.316 | -0.077 | -0.256 | 0.101 | 0.393 |
| Middle temporal | -0.259 | -0.415 | -0.104 | 0.001* | -0.238 | -0.413 | -0.063 | 0.008* |
| Paracentral | -0.089 | -0.236 | 0.058 | 0.234 | -0.053 | -0.211 | 0.105 | 0.512 |
| Parahippocampal | -0.092 | -0.254 | 0.069 | 0.260 | -0.120 | -0.310 | 0.070 | 0.213 |
| Pars opercularis | -0.220 | -0.369 | -0.070 | 0.004* | -0.184 | -0.348 | -0.020 | 0.028 |
| Pars orbitalis | -0.089 | -0.245 | 0.067 | 0.263 | -0.053 | -0.228 | 0.123 | 0.554 |
| Pars triangularis | -0.164 | -0.312 | -0.016 | 0.030 | -0.127 | -0.290 | 0.036 | 0.125 |
| Pericalcarine | -0.086 | -0.221 | 0.050 | 0.215 | -0.036 | -0.202 | 0.130 | 0.672 |
| Postcentral | -0.228 | -0.375 | -0.082 | 0.002* | -0.183 | -0.344 | -0.022 | 0.026 |
| Posterior cingulate | -0.169 | -0.319 | -0.019 | 0.028 | -0.158 | -0.324 | 0.007 | 0.060 |
| Precentral | -0.199 | -0.337 | -0.061 | 0.005* | -0.169 | -0.327 | -0.011 | 0.036 |
| Precuneus | -0.266 | -0.415 | -0.117 | < 0.001* | -0.251 | -0.414 | -0.087 | 0.003* |
| Rostral anterior cingulate | -0.052 | -0.216 | 0.111 | 0.529 | -0.023 | -0.200 | 0.154 | 0.796 |
| Rostral middle frontal | -0.222 | -0.371 | -0.073 | 0.004* | -0.185 | -0.348 | -0.023 | 0.025 |
| Superior frontal | -0.146 | -0.300 | 0.008 | 0.064 | -0.108 | -0.280 | 0.063 | 0.213 |
| Superior parietal | -0.256 | -0.401 | -0.111 | < 0.001* | -0.242 | -0.407 | -0.076 | 0.004* |
| Superior temporal | -0.218 | -0.376 | -0.061 | 0.007* | -0.180 | -0.356 | -0.004 | 0.045 |
| Supramarginal | -0.310 | -0.459 | -0.160 | < 0.001* | -0.273 | -0.442 | -0.103 | 0.002* |
| Temporal pole | -0.067 | -0.238 | 0.105 | 0.443 | -0.095 | -0.291 | 0.101 | 0.337 |
| Transverse temporal | -0.074 | -0.222 | 0.074 | 0.324 | -0.048 | -0.209 | 0.112 | 0.552 |
| Frontal | -0.285 | -0.439 | -0.131 | < 0.001* | -0.229 | -0.397 | -0.061 | 0.008* |
| Temporal | -0.088 | -0.243 | 0.068 | 0.267 | -0.066 | -0.234 | 0.102 | 0.439 |
| Occipital | -0.229 | -0.374 | -0.084 | 0.002* | -0.208 | -0.371 | -0.046 | 0.012 |
| Parietal | -0.165 | -0.301 | -0.029 | 0.018* | -0.128 | -0.278 | 0.023 | 0.097 |

**Supplementary Table 4 – Longitudinal Associations between Changes in Regional Partial volume corrected Cerebral Blood Flow and Changes in Tau Levels.** Banks STS - Banks of the superior temporal sulcus; CI – Confidence Interval; CI lower – Lower bound of the 95% Confidence Interval; CI upper – Upper bound of the 95% Confidence Interval. * indicates associations that survived false discovery rate correction.

|  | **Adjusting for age, sex, APOE ε4, hypertension and regional atrophy** | | | | **Further adjusting for Aβ** | | | |
| --- | --- | --- | --- | --- | --- | --- | --- | --- |
| Regions | **β** | **CI lower** | **CI upper** | **P value** | **β** | **CI lower** | **CI upper** | **P value** |
| Banks STS | -0.179 | -0.384 | 0.027 | 0.088 | -0.167 | -0.388 | 0.055 | 0.139 |
| Caudal anterior cingulate | 0.059 | -0.191 | 0.310 | 0.639 | 0.110 | -0.158 | 0.378 | 0.416 |
| Caudal middle frontal | -0.207 | -0.405 | -0.008 | 0.041 | -0.180 | -0.394 | 0.035 | 0.100 |
| Cuneus | -0.145 | -0.314 | 0.024 | 0.092 | -0.123 | -0.314 | 0.067 | 0.202 |
| Entorhinal | -0.126 | -0.306 | 0.054 | 0.168 | -0.184 | -0.393 | 0.025 | 0.085 |
| Frontal pole | -0.014 | -0.232 | 0.205 | 0.903 | -0.011 | -0.242 | 0.220 | 0.923 |
| Fusiform | -0.256 | -0.461 | -0.051 | 0.015 | -0.295 | -0.529 | -0.062 | 0.014 |
| Inferior parietal | -0.317 | -0.522 | -0.112 | 0.003* | -0.298 | -0.522 | -0.074 | 0.010 |
| Inferior temporal | -0.222 | -0.446 | 0.002 | 0.052 | -0.197 | -0.449 | 0.055 | 0.125 |
| Isthmus cingulate | -0.333 | -0.529 | -0.137 | 0.001* | -0.323 | -0.538 | -0.107 | 0.004 |
| Lateral occipital | -0.253 | -0.439 | -0.067 | 0.008 | -0.311 | -0.541 | -0.082 | 0.008 |
| Lateral orbitofrontal | -0.015 | -0.234 | 0.203 | 0.889 | 0.007 | -0.228 | 0.242 | 0.954 |
| Lingual | -0.081 | -0.252 | 0.091 | 0.353 | -0.079 | -0.286 | 0.128 | 0.451 |
| Medial orbitofrontal | -0.029 | -0.270 | 0.212 | 0.814 | -0.011 | -0.269 | 0.247 | 0.935 |
| Middle temporal | -0.212 | -0.448 | 0.023 | 0.077 | -0.177 | -0.440 | 0.085 | 0.183 |
| Paracentral | 0.007 | -0.216 | 0.230 | 0.951 | 0.052 | -0.188 | 0.291 | 0.671 |
| Parahippocampal | -0.163 | -0.370 | 0.043 | 0.119 | -0.206 | -0.447 | 0.034 | 0.091 |
| Pars opercularis | -0.133 | -0.336 | 0.070 | 0.196 | -0.122 | -0.339 | 0.095 | 0.268 |
| Pars orbitalis | -0.019 | -0.238 | 0.200 | 0.863 | -0.001 | -0.231 | 0.229 | 0.994 |
| Pars triangularis | -0.094 | -0.298 | 0.110 | 0.364 | -0.090 | -0.307 | 0.128 | 0.417 |
| Pericalcarine | -0.090 | -0.251 | 0.070 | 0.269 | -0.066 | -0.272 | 0.140 | 0.529 |
| Postcentral | -0.209 | -0.440 | 0.023 | 0.077 | -0.164 | -0.420 | 0.091 | 0.205 |
| Posterior cingulate | -0.162 | -0.377 | 0.053 | 0.138 | -0.120 | -0.357 | 0.117 | 0.318 |
| Precentral | -0.112 | -0.311 | 0.086 | 0.263 | -0.077 | -0.295 | 0.141 | 0.488 |
| Precuneus | -0.254 | -0.459 | -0.048 | 0.016 | -0.242 | -0.465 | -0.020 | 0.033 |
| Rostral anterior cingulate | 0.099 | -0.156 | 0.353 | 0.443 | 0.117 | -0.145 | 0.379 | 0.378 |
| Rostral middle frontal | -0.115 | -0.326 | 0.095 | 0.280 | -0.112 | -0.337 | 0.114 | 0.330 |
| Superior frontal | -0.052 | -0.269 | 0.165 | 0.634 | -0.031 | -0.265 | 0.204 | 0.796 |
| Superior parietal | -0.292 | -0.482 | -0.102 | 0.003* | -0.266 | -0.482 | -0.050 | 0.016 |
| Superior temporal | -0.115 | -0.340 | 0.110 | 0.314 | -0.097 | -0.339 | 0.145 | 0.427 |
| Supramarginal | -0.274 | -0.490 | -0.058 | 0.013 | -0.250 | -0.488 | -0.013 | 0.039 |
| Temporal pole | -0.201 | -0.421 | 0.020 | 0.074 | -0.245 | -0.495 | 0.006 | 0.056 |
| Transverse temporal | 0.048 | -0.155 | 0.251 | 0.640 | 0.036 | -0.173 | 0.245 | 0.731 |
| Frontal | -0.179 | -0.384 | 0.027 | 0.088 | -0.167 | -0.388 | 0.055 | 0.139 |
| Temporal | 0.059 | -0.191 | 0.310 | 0.639 | 0.110 | -0.158 | 0.378 | 0.416 |
| Occipital | -0.207 | -0.405 | -0.008 | 0.041 | -0.180 | -0.394 | 0.035 | 0.100 |
| Parietal | -0.145 | -0.314 | 0.024 | 0.092 | -0.123 | -0.314 | 0.067 | 0.202 |

**Supplementary Table 5 – Cross-sectional associations between regional CBF and tau levels after additional adjustment for regional brain atrophy.**
Banks STS- Banks of the superior temporal sulcus; CI – Confidence Interval; CI lower – Lower bound of the 95% Confidence Interval; CI upper – Upper bound of the 95% Confidence Interval. * indicates associations that survived false discovery rate correction.

|  | **Adjusting for age, sex, APOE ε4, hypertension and regional atrophy** | | | | **Further adjusting for Aβ** | | | |
| --- | --- | --- | --- | --- | --- | --- | --- | --- |
| Regions | **β** | **CI lower** | **CI upper** | **P value** | **β** | **CI lower** | **CI upper** | **P value** |
| Banks STS | -0.243 | -0.402 | -0.084 | 0.003* | -0.229 | -0.397 | -0.061 | 0.008* |
| Caudal anterior cingulate | -0.017 | -0.161 | 0.127 | 0.812 | -0.066 | -0.234 | 0.102 | 0.439 |
| Caudal middle frontal | -0.237 | -0.387 | -0.087 | 0.002* | -0.208 | -0.371 | -0.046 | 0.012 |
| Cuneus | -0.183 | -0.319 | -0.046 | 0.009* | -0.128 | -0.278 | 0.023 | 0.097 |
| Entorhinal | -0.137 | -0.293 | 0.019 | 0.086 | -0.142 | -0.333 | 0.050 | 0.145 |
| Frontal pole | -0.015 | -0.174 | 0.144 | 0.852 | -0.024 | -0.206 | 0.158 | 0.793 |
| Fusiform | -0.228 | -0.385 | -0.072 | 0.005* | -0.196 | -0.383 | -0.010 | 0.039 |
| Inferior parietal | -0.352 | -0.509 | -0.195 | < 0.001* | -0.298 | -0.461 | -0.134 | < 0.001* |
| Inferior temporal | -0.187 | -0.350 | -0.024 | 0.025* | -0.193 | -0.378 | -0.009 | 0.040 |
| Isthmus cingulate | -0.275 | -0.426 | -0.124 | < 0.001* | -0.203 | -0.366 | -0.039 | 0.015 |
| Lateral occipital | -0.240 | -0.390 | -0.089 | 0.002* | -0.225 | -0.403 | -0.047 | 0.014 |
| Lateral orbitofrontal | -0.076 | -0.229 | 0.077 | 0.329 | -0.100 | -0.275 | 0.076 | 0.264 |
| Lingual | -0.115 | -0.255 | 0.026 | 0.109 | -0.067 | -0.236 | 0.101 | 0.432 |
| Medial orbitofrontal | -0.069 | -0.224 | 0.086 | 0.383 | -0.077 | -0.256 | 0.101 | 0.393 |
| Middle temporal | -0.249 | -0.420 | -0.078 | 0.005* | -0.238 | -0.413 | -0.063 | 0.008* |
| Paracentral | -0.071 | -0.219 | 0.077 | 0.343 | -0.053 | -0.211 | 0.105 | 0.512 |
| Parahippocampal | -0.183 | -0.341 | -0.024 | 0.025* | -0.120 | -0.310 | 0.070 | 0.213 |
| Pars opercularis | -0.213 | -0.365 | -0.061 | 0.006* | -0.184 | -0.348 | -0.020 | 0.028 |
| Pars orbitalis | -0.041 | -0.203 | 0.121 | 0.617 | -0.053 | -0.228 | 0.123 | 0.554 |
| Pars triangularis | -0.142 | -0.294 | 0.010 | 0.068 | -0.127 | -0.290 | 0.036 | 0.125 |
| Pericalcarine | -0.139 | -0.272 | -0.006 | 0.040 | -0.036 | -0.202 | 0.130 | 0.672 |
| Postcentral | -0.224 | -0.374 | -0.074 | 0.004* | -0.183 | -0.344 | -0.022 | 0.026 |
| Posterior cingulate | -0.157 | -0.309 | -0.005 | 0.043 | -0.158 | -0.324 | 0.007 | 0.060 |
| Precentral | -0.173 | -0.314 | -0.033 | 0.016* | -0.169 | -0.327 | -0.011 | 0.036 |
| Precuneus | -0.296 | -0.453 | -0.140 | < 0.001* | -0.251 | -0.414 | -0.087 | 0.003* |
| Rostral anterior cingulate | -0.019 | -0.175 | 0.137 | 0.810 | -0.023 | -0.200 | 0.154 | 0.796 |
| Rostral middle frontal | -0.187 | -0.344 | -0.031 | 0.019* | -0.185 | -0.348 | -0.023 | 0.025 |
| Superior frontal | -0.115 | -0.272 | 0.042 | 0.150 | -0.108 | -0.280 | 0.063 | 0.213 |
| Superior parietal | -0.305 | -0.454 | -0.156 | < 0.001* | -0.242 | -0.407 | -0.076 | 0.004* |
| Superior temporal | -0.182 | -0.349 | -0.014 | 0.034 | -0.180 | -0.356 | -0.004 | 0.045 |
| Supramarginal | -0.300 | -0.454 | -0.145 | < 0.001* | -0.273 | -0.442 | -0.103 | 0.002* |
| Temporal pole | -0.151 | -0.317 | 0.015 | 0.074 | -0.095 | -0.291 | 0.101 | 0.337 |
| Transverse temporal | -0.007 | -0.153 | 0.139 | 0.925 | -0.048 | -0.209 | 0.112 | 0.552 |
| Frontal | -0.243 | -0.402 | -0.084 | 0.003* | -0.229 | -0.397 | -0.061 | 0.008* |
| Temporal | -0.017 | -0.161 | 0.127 | 0.812 | -0.066 | -0.234 | 0.102 | 0.439 |
| Occipital | -0.237 | -0.387 | -0.087 | 0.002* | -0.208 | -0.371 | -0.046 | 0.012 |
| Parietal | -0.183 | -0.319 | -0.046 | 0.009* | -0.128 | -0.278 | 0.023 | 0.097 |

**Supplementary Table 6– Longitudinal associations between changes in regional CBF and changes in tau levels after additional adjustment for regional brain atrophy.** Banks STS - Banks of the superior temporal sulcus; CI – Confidence Interval; CI lower – Lower bound of the 95% Confidence Interval; CI upper – Upper bound of the 95% Confidence Interval. * indicates associations that survived false discovery rate correction.
