# Supplementary material for: The Associations of cerebral blood flow and white matter hyperintensities with tau and amyloid-beta across the Alzheimer’s disease spectrum
Source: Brain Imaging Behav. 2026 Aug 5;20(4):117. doi: 10.1007/s11682-026-01187-x (PMC13442569; doi:10.1007/s11682-026-01187-x)
Supplement: Supplementary file 2 [file 11682_2026_1187_MOESM2_ESM.docx]

**Supplementary Figure 1: Associations between cerebral blood flow (CBF) and Amyloid-β (Aβ) levels.**

**(a):** cross-sectional associations between CBF and Aβ at baseline. **(b):** cross-sectional associations between CBF and Aβ levels at baseline after accounting for meta temporal tau levels. **(c)** Within-subject effects in the longitudinal associations between changes in CBF and Aβ over time, after adjusting for baseline age, sex, hypertension and APOE ε4, using a linear mixed-effects model with random intercept. **(d)** Within-subject effects in the longitudinal associations between changes in CBF and Aβ after further accounting for **tau** changes. For the beta (estimate) maps (left panels), colours represent the direction and magnitude of the association: red indicates positive effects, and blue indicates negative effects, only regions that p-value larger than 0.08 were coloured (non-significance). For the p-value maps (right panels), colours reflect statistical significance, with red denoting lower p-values.
