# Supplementary material for: The Associations of cerebral blood flow and white matter hyperintensities with tau and amyloid-beta across the Alzheimer’s disease spectrum
Source: Brain Imaging Behav. 2026 Aug 5;20(4):117. doi: 10.1007/s11682-026-01187-x (PMC13442569; doi:10.1007/s11682-026-01187-x)
Supplement: Supplementary file 3 [file 11682_2026_1187_MOESM3_ESM.docx]

Supplementary Text

# Comparing CBF derived from ventricular CSF calibration, N4 bias field correction + ventricular CSF calibration, and voxel-wise calibration

A subset of 16 participants, including 8 CN and 8 MCI participants, with prominent posterior hyperintensity on the baseline PWI data were selected for the comparison. It is noticed that individuals with posterior bias in the baseline PWI also had the bias in follow-up scans. We compared three calibration/correction approaches: 1) calibrating using CSF signal from ventricular mask, 2) estimating low frequency bias field by applying ANTs’ N4BiasFieldCorrection to M0 scan and correcting for the same bias field in PWI. Ventricular CSF calibration (same as the first approach) was then applied to bias-field-corrected PWI, and 3) using voxel-wise calibration available in FSL BASIL.

Visual inspection showed that the voxel-wise calibration approach produced the greatest reduction in regional brightness variation and the most homogeneous intensity profile across the brain. Application of N4 bias field correction before ventricular CSF calibration also visibly reduced posterior hyperintensity, whereas ventricular CSF calibration alone preserved the regional intensity pattern because it applies only a global scaling factor. The visual appearance of the N4-corrected images remained less homogeneous than that obtained with voxel-wise calibration.

We then examined differences in total grey matter CBF derived from each of these three approaches between diagnostic groups (MCI vs. CN). In the baseline diagnosis model, total grey matter CBF using ventricular CSF calibration was lower in MCI compared with that in CN (β = −3.02, 95% CI [−5.97, -0.07], p = 0.048). The voxelwise method showed a similar direction of effect (β = −4.88, 95% CI [−14.75, 5.01], p = 0.306). In contrast, CBF quantified with N4 bias field correction and ventricular CSF calibration did not show significant differences between diagnostic groups (β = 4.24, 95% CI [−24.70, 33.18], p = 0.753).

Across all three processing protocols, there was no significant difference in longitudinal CBF change between MCI and CN participants. Specifically, the Time × MCI interaction was not significant for the ventricular CSF calibration method (β = 0.105, 95% CI [−0.062, 0.273], p = 0.213), the N4 correction + ventricular CSF calibration method (β = −0.489, 95% CI [−1.312, 0.340], p = 0.240), or the voxel-wise method (β = 0.107, 95% CI [−0.315, 0.528], p = 0.615).

Total grey matter CBF derived from ventricular CSF calibration significantly declined over time (β = −0.130, 95% CI [−0.215, −0.045], p = 0.003). Total grey matter CBF using N4 correction + ventricular CSF calibration also showed significant Time effect (β = −0.655, 95% CI [−1.070, −0.240], p = 0.003). The voxel-wise method did not reach statistical significance (β = -0.086, 95% CI [−0.188, 0.015], p = 0.095).

Age was not significantly associated with total grey matter CBF across the three processing protocols. In the ventricular CSF calibration method, age showed a trend-level negative association with total grey matter CBF (β = −0.310, 95% CI [−0.640, 0.020], p = 0.065). A similar trend-level negative association was observed using the voxel-wise method (β = −0.363, 95% CI [−0.759, 0.034], p = 0.072). In contrast, the N4 correction + ventricular CSF calibration method showed no evidence of an age effect (β = 0.097, 95% CI [−2.474, 2.669], p = 0.937).

In conclusion, the ventricle CSF calibration method provided visually acceptable intensity homogeneity, preserved biologically plausible diagnosis-related differences, and showed an expected longitudinal decline in total grey matter CBF. Therefore, we used this approach in the main text.

# Associations of CBF with cognition, demographics, and Time

At baseline, total grey matter (GM) CBF was significantly lower in older individuals (β = -0.188, p = 0.001), after controlling for sex, and in males compared to females (mean difference, 5.330 mL/100g/min; p < 0.001), adjusting for age. Total GM CBF significantly differed between classification groups (p = 0.004; CN, 35.0 ± 8.54 mL/100g/min; MCI, 30.7 ± 9.58 mL/100g/min; AD, 25.8 ± 9.76 mL/100g/min). All lobar CBF also significantly differed between classification groups (all p < 0.001). Post-hoc analysis showed significantly lower total GM and lobar CBF (mean difference = -9.24 - -6.14, p = 0.001–0.004) in AD compared to those in CN. Higher total GM and lobar CBF were associated with significantly higher MMSE, MoCA and CDR memory scores (β = 0.156 – 0.207, p < 0.009). Total GM CBF, and CBF in all four lobes, caudate, and thalamus, significantly declined over time (β = -0.148 - -0.086, p < 0.007). Total white matter (WM) CBF was significantly lower in males compared to females (mean difference, 4.19 mL/100g/min; p < 0.001) after adjusting for age. Additionally, WM CBF was significantly lower in AD compared to CN (mean difference, 6.00 mL/100g/min; p = 0.001). There was no significant difference in CBF between WMH and NAWM (p = 0.678).

# Associations of WMH with cognition, demographics, and Time

At baseline, WBWMH, PVWMH, and DWMH volumes were higher in older participants (β = 0.360 - 0.409, p < 0.001), but there were no sex differences. There was a marginal association between WBWMH volumes and classification (β = 0.192, p = 0.060), with post-hoc analysis suggesting that WBWMH was marginally higher in AD than CN (β = 0.455, p = 0.055). There were no significant associations between WMH and CBF after adjusting for age and sex (p = 0.085 – 0.523), or between WMH volumes and neuropsychological test scores after controlling for age, sex and education (p = 0.125 – 0.529). WBWMH (β = 0.080, p < 0.001) and PVWMH (β = 0.060, p = 0.010) volumes significantly increased over time, while DWMH showed a marginal increase (β = 0.047, p = 0.088). A significant interaction between Time and classification group was observed for DWMH progression (p = 0.042). Stratified analysis showed that MCI exhibited a significantly faster increase in DWMH volumes over time (β = 0.006, p = 0.014), compared to CN.

# Sensitivity test with partial volume corrected CBF

Additional sensitivity analyses were conducted using partial volume corrected CBF estimates to evaluate whether the observed tau–CBF associations were influenced by partial volume effects in the CBF maps.

In the cross-sectional partial volume corrected analyses, after adjusting for age, sex, hypertension, and APOE ε4 status, higher tau levels were significantly associated with lower CBF after FDR correction in the caudal middle frontal, inferior parietal, isthmus cingulate, middle temporal, precuneus, superior parietal, and supramarginal regions (Supplementary Table 3). After further adjustment for Aβ, no regional associations survived FDR correction (Supplementary Table 3). This pattern was broadly consistent with the findings reported in the main text.

In the longitudinal analyses of partial volume corrected CBF, after FDR correction, significant negative associations between within-subject changes in tau and CBF were observed in the banks of the superior temporal sulcus, caudal middle frontal, cuneus, inferior parietal, isthmus cingulate, lateral occipital, middle temporal, pars opercularis, postcentral, precentral, precuneus, rostral middle frontal, superior parietal, superior temporal, and supramarginal regions (Supplementary Table 4). After further adjustment for changes in Aβ, significant associations remained in the banks of the superior temporal sulcus, inferior parietal, middle temporal, precuneus, superior parietal, and supramarginal regions (Supplementary Table 4).

# Sensitivity test adjusting for regional brain atrophy

Additional sensitivity analyses were performed to examine whether the tau–CBF associations persisted after further accounting for regional brain atrophy. In the cross-sectional analyses, nominally significant negative associations between regional CBF and tau were observed in several frontal, temporal, parietal, occipital, and cingulate regions after adjusting for age, sex, APOE ε4 status, hypertension category, and regional atrophy. After FDR correction, these associations remained significant in the inferior parietal, isthmus cingulate, and superior parietal regions. After further adjustment for Aβ, nominal associations persisted in several regions, but none survived FDR correction.

In the longitudinal analyses, nominally significant negative associations between within-subject changes in CBF and tau were observed across widespread cortical regions after adjustment for age, sex, APOE ε4 status, hypertension category, and regional atrophy. After FDR correction, these associations remained significant in the banks of the superior temporal sulcus, caudal middle frontal, cuneus, fusiform, inferior parietal, inferior temporal, isthmus cingulate, lateral occipital, middle temporal, parahippocampal, pars opercularis, postcentral, precentral, precuneus, rostral middle frontal, superior parietal, supramarginal, frontal, and occipital regions. After further adjustment for Aβ changes, FDR-significant associations remained in the banks of the superior temporal sulcus, inferior parietal, middle temporal, precuneus, superior parietal, supramarginal, and frontal regions. Overall, these results were broadly consistent with the main analyses. The spatial pattern was largely similar, although some regions reported in the main text, such as lateral occipital, did not remain significant after further adjustment for Aβ changes, while the frontal composite remained FDR-significant in the atrophy-adjusted model.

# Participating IRBs and ethics committees

The Ethics committees/institutional review boards that approved the ADNI study are: Albany Medical Center Committee on Research Involving Human Subjects Institutional Review Board, Boston University Medical Campus and Boston Medical Center Institutional Review Board, Butler Hospital Institutional Review Board, Cleveland Clinic Institutional Review Board, Columbia University Medical Center Institutional Review Board, Duke University Health System Institutional Review Board, Emory Institutional Review Board, Georgetown University Institutional Review Board, Health Sciences Institutional Review Board, Houston Methodist Institutional Review Board, Howard University Office of Regulatory Research Compliance, Icahn School of Medicine at Mount Sinai Program for the Protection of Human Subjects, Indiana University Institutional Review Board, Institutional Review Board of Baylor College of Medicine, Jewish General Hospital Research Ethics Board, Johns Hopkins Medicine Institutional Review Board, Lifespan - Rhode Island Hospital Institutional Review Board, Mayo Clinic Institutional Review Board, Mount Sinai Medical Center Institutional Review Board, Nathan Kline Institute for Psychiatric Research & Rockland Psychiatric Center Institutional Review Board, New York University Langone Medical Center School of Medicine Institutional Review Board, Northwestern University Institutional Review Board, Oregon Health and Science University Institutional Review Board, Partners Human Research Committee Research Ethics, Board Sunnybrook Health Sciences Centre, Roper St. Francis Healthcare Institutional Review Board, Rush University Medical Center Institutional Review Board, St. Joseph's Phoenix Institutional Review Board, Stanford Institutional Review Board, The Ohio State University Institutional Review Board, University Hospitals Cleveland Medical Center Institutional Review Board, University of Alabama Office of the IRB, University of British Columbia Research Ethics Board, University of California Davis Institutional Review Board Administration, University of California Los Angeles Office of the Human Research Protection Program, University of California San Diego Human

Research Protections Program, University of California San Francisco Human Research Protection Program, University of Iowa Institutional Review Board, University of Kansas Medical Center Human Subjects Committee, University of Kentucky Medical Institutional Review Board, University of Michigan Medical School Institutional Review Board, University of Pennsylvania Institutional Review Board, University of Pittsburgh Institutional Review Board, University of Rochester Research Subjects Review Board, University of South Florida Institutional Review Board, University of Southern, California Institutional Review Board, UT Southwestern Institution Review Board, VA Long Beach Healthcare System Institutional Review Board, Vanderbilt University Medical Center Institutional Review Board, Wake Forest School of Medicine Institutional Review Board, Washington University School of Medicine Institutional Review Board, Western Institutional Review Board, Western University Health Sciences Research Ethics Board, and Yale University Institutional Review Board.

**Supplementary Text Figure 1. Visual comparison of CBF maps generated using three correction approaches.**
Baseline perfusion-weighted images (PWIs) are shown for representative participants selected for prominent posterior hyperintensity. Each column represents one participant, and axial slices were selected at approximately comparable anatomical levels across rows. Rows from top to bottom show: **original PWI**, **ventricular CSF calibration**, **N4 + ventricular CSF calibration**, and **voxel-wise calibration**. The original PWI images showed visible regional hyperintensity. Ventricular CSF calibration preserved this regional intensity pattern, whereas N4 bias field correction followed by ventricular CSF calibration reduced posterior hyperintensity. Voxel-wise calibration produced the most homogeneous intensity profile across the brain.

Supplementary Text Table 1. Sensitivity analysis comparing total grey matter CBF estimates across calibration and bias correction methods.

| Method | diagnosis | Total grey matter CBF |
| --- | --- | --- |
| Ventricle mask | CN | 41.7 ± 8.37 |
| Ventricle mask | MCI | 34.8 ± 7.68 |
| Ventricle mask + N4bias field correction | CN | 58.1 ± 18.8 |
| Ventricle mask + N4bias field correction | MCI | 72.5 ± 11.9 |
| Oxford_asl Voxelwise | CN | 48.9 ± 9.15 |
| Oxford_asl Voxelwise | MCI | 44.3 ± 7.15 |

Values are presented as mean ± standard deviation for total grey matter CBF in the selected sensitivity subset. This subset included 16 participants, comprising 8 CN and 8 MCI participants with visually prominent posterior hyperintensity on baseline perfusion-weighted images. Three processing approaches were compared: ventricular CSF calibration, ventricular CSF calibration after N4 bias field correction, and Oxford_ASL voxel-wise calibration. The ventricular CSF calibration and voxel-wise calibration approaches showed lower mean total grey matter CBF in MCI than CN participants, whereas the N4 bias field correction + ventricular CSF calibration approach showed greater variability and a reversed group mean pattern.
